# Associação da Hipertensão com a Gravidade e a Mortalidade de Pacientes Hospitalizados com COVID-19 em Wuhan, China: Estudo Unicêntrico e Retrospectivo

**DOI:** 10.36660/abc.20200733

**Published:** 2021-07-07

**Authors:** You-ping Deng, Wen Xie, Tao Liu, Shou-yi Wang, Mei-rong Wang, Yu-xing Zan, Xiao-bo Meng, Yu-qing Deng, Hai-rong Xiong, Xue-dong Fu

**Affiliations:** 1 Department of Pediatrics Zhongnan Hospital Wuhan University Wuhan China Department of Pediatrics, Zhongnan Hospital of Wuhan University, Wuhan - China; 2 Department of Clinical Laboratory Zhongnan Hospital Wuhan University Wuhan China Department of Clinical Laboratory, Zhongnan Hospital of Wuhan University, Wuhan - China; 3 Department of Urology Zhongnan Hospital Wuhan University Wuhan China Department of Urology, Zhongnan Hospital of Wuhan University, Wuhan - China; 4 Institute of Medical Virology School of Basic Medical Sciences Wuhan University Wuhan China State Key Laboratory of Virology/ Institute of Medical Virology, School of Basic Medical Sciences, Wuhan University, Wuhan - China

**Keywords:** COVID-19/complicações, Betacoronavírus, Síndrome Respiratória Aguda Grave, Hipertensão, Comorbidades, Fatores de Risco

## Abstract

**Fundamento:**

A doença Coronavírus 2019 (COVID-19), causada pela síndrome respiratória aguda grave Coronavírus 2 (SARS-CoV-2), espalhou-se pelo mundo.

**Objetivo:**

Investigar a associação entre a hipertensão e a gravidade/mortalidade de pacientes hospitalizados com COVID-19 em Wuhan, China.

**Métodos:**

Um total de 337 pacientes diagnosticados com COVID-19 no Sétimo Hospital da cidade de Wuhan, de 20 de janeiro a 25 de fevereiro de 2020, foram inseridos e analisados em um estudo de caso unicêntrico e retrospectivo. O nível de significância adotado para a análise estatística foi 0,05.

**Resultados:**

Dos 337 pacientes com diagnóstico confirmado de COVID-19, 297 (87.8%) tiveram alta do hospital e 40 pacientes (22,9%) morreram. A idade média foi de 58 anos (variando de 18 a 91 anos). Havia 112 (33,2%) pacientes diagnosticados com hipertensão no momento da internação (idade média, 65,0 anos [variação, 38-91 anos]; sendo 67 homens [59,8%, IC95%: 50,6%-69,0%], p=0,0209). Pacientes com hipertensão apresentaram uma porção significativamente maior de casos graves (69 [61,6%, IC95%: 52,5%-70,8%] vs. 117 [52,0%, IC95%: 45,4%-58,6%] em pacientes graves e 23 [19,3%, IC95%: 12,9%-28,1%] vs. 27 [12,0%, IC95%: 7,7%-16,3%] em pacientes críticos, p=0,0014) e maiores taxas de mortalidade (20 [17,9%, IC95%: 10,7%-25,1%] vs. 20 [8,9%, IC95%: 5,1%-12,6%, p=0,0202). Além disso, pacientes hipertensos apresentaram níveis anormais de vários indicadores, como linfopenia e inflamação, e nas funções cardíacas, hepáticas, renais e pulmonares no momento da internação. O grupo de pacientes com hipertensão também demonstrou níveis maiores de TNT e creatinina próximo da alta.

**Conclusão:**

A hipertensão está altamente associada à gravidade ou mortalidade da COVID-19. Um tratamento agressivo deve ser considerado para pacientes hipertensos com COVID-19, principalmente com relação a lesões cardíacas e dos rins.

## Introdução

A nova doença Coronavírus 2019 (COVID-19), causada pela síndrome respiratória aguda grave Coronavírus 2 (SARS-CoV-2), surgiu em Wuhan em dezembro de 2019 e se espalhou pelo mundo, gerando grande preocupação para a economia e a saúde pública global.^1^ A SARS-CoV-2 foi identificada como o patógeno da COVID-19 em janeiro de 2020, e pertence a um clado do subgênero Sarbecovirus, da subfamília Orthocoronavirinae.^2^ Este novo coronavírus é um vírus envelopado constituído por RNA de cadeia simples e senso positivo, e reconhece a enzima conversora da angiotensina 2 (ECA2) como o receptor funcional de entrada da célula. A ECA2 é membro da família da enzima conversora da angiotensina (ECA) e tem papel importante nas funções fisiológicas do ser humano, especialmente na regulação da pressão arterial.^3 , 4^ Dados recentes reportaram os aspectos clínicos gerais e as características epidemiológicas de pacientes com COVID-19, e muitos relatos demonstraram que a lesão cardíaca está associada ao maior risco de mortalidade em pacientes com COVID-19.^5 - 7^

Há alta prevalência de hipertensão pelo mundo, principalmente na China. No geral, a hipertensão esteve presente em 23,2% da população chinesa adulta de 2012 a 2015.^8^ A hipertensão é um fator de risco importante para doenças cardiovasculares, a principal causa de morte na China.^9 , 10^ Com a urbanização, o aumento da renda e a população mais velha, a carga de hipertensão de doenças cardiovasculares está crescendo na China.^11 , 12^ Evidências sugerem que a hipertensão pode estar relacionada a um crescente fator de mortalidade hospitalar devido à COVID-19.^13 , 14^ Assim, iniciamos este estudo retrospectivo para analisar dados de um centro em Wuhan, China, e examinar a associação entre hipertensão e COVID-19. Também monitoramos as mudanças dinâmicas de importantes biomarcadores entre os pacientes hospitalizados, o que pode trazer recomendações para o manejo clínico de pacientes hipertensos com COVID-19.

## Métodos

### Inclusão dos pacientes

O Comitê de Ética Institucional do Hospital Zhongnan, da Universidade de Wuhan, aprovou este projeto (n.2020056K). O Sétimo Hospital de Wuhan foi um dos primeiros hospitais designados para a COVID-19, e foi consignado ao Hospital de Zhongnan, da Universidade de Wuhan, desde janeiro de 2020. Um total de 337 pacientes com diagnóstico confirmado de COVID-19 hospitalizados em quatro enfermarias do Sétimo Hospital de Wuhan foram incluídos no estudo, realizado de 20 de janeiro a 25 de fevereiro de 2020. Todos os pacientes foram diagnosticados com COVID-19 e classificados em tipos clínicos diferentes, de acordo com as diretrizes diagnósticas e de tratamento da COVID-19 da Comissão Nacional de Saúde Chinesa (versão 3-7).^15^ Como os pacientes com mais complicações foram enviados a hospitais móveis de campanha devido à hierarquia do sistema médico durante o início da pandemia, todos os pacientes envolvidos neste estudo tinham casos moderados (101), graves (186) e críticos (50) da COVID-19. Os casos críticos foram transferidos para a UTI. As amostras de *swab* da garganta foram coletadas e enviadas para a detecção no laboratório.

### Coleta de dados

Os registros médicos, incluindo informações básicas (idade, gênero, comorbidades etc.), tratamento e resultados de cada paciente com resultados positivos para SARS-CoV-2 foram coletados. A data do início da doença foi especificada como o dia em que os sintomas foram observados. Os resultados clínicos foram avaliados e registrados no momento da alta ou transferência para a unidade de terapia intensiva. A confirmação laboratorial de SARS-CoV-2 foi majoritariamente realizada no laboratório clínico do Hospital Zhongnan, da Universidade de Wuhan, e parcialmente no laboratório clínico do Sétimo Hospital de Wuhan após o sistema de detecção ter sido localmente estabelecido desde o fim de fevereiro. A SARS-CoV-2 foi verificada em tempo real utilizando o teste RT-PCR, com o protocolo já descrito previamente.^16^ A detecção dos ácidos nucleicos virais com a amostra de *swab* da garganta foi realizada durante o processo terapêutico. Além disso, as amostras de pacientes também mostraram outras infecções, como o vírus influenza, parainfluenza, Coxsackie, adenovírus, ecovírus, vírus sincicial respiratório, citomegalovírus etc. Todos os pacientes realizaram tomografia computadorizada ou raio-x. Um exame radiológico de acompanhamento e o teste negativo para SARS-CoV-2 foram considerados como critérios para cura e alta hospitalar.

As manifestações clínicas foram resumidas, incluindo febre, tosse, expectoração, mialgia, fadiga, dor de cabeça, palpitações cardíacas, diarreia, dispneia etc. Os exames laboratoriais foram realizados na internação e conforme a progressão da doença, como exames de sangue de rotina, bioquímica sanguínea, concentração de gases no sangue, eletrólitos do sangue, coagulação, procalcitonina (PCT), proteína C reativa (PCR), soro amiloide A (SAA), creatina quinase e enzima do miocárdio. Os tratamentos médicos foram registrados, já que a maioria dos pacientes recebeu o tratamento antiviral ou uma mediação patenteada da China. Os pacientes também receberam corticosteroides, gama-globulina, probióticos ou assistência respiratória quando necessário.

### Análise estatística

Dados categóricos foram apresentados como frequência e porcentagem, e dados contínuos foram descritos usando mediana e intervalo interquartil (IIQ). As variáveis contínuas foram testadas pela distribuição gaussiana utilizando o teste de normalidade D’Agostino-Pearson; depois, foram analisados com o teste de Mann-Whitney, quando apropriado. As frequências das variáveis categóricas foram comparadas com o teste Qui-quadrado, com o teste exato de Fisher e o teste de Kruskal-Wallis, quando apropriado. As curvas de sobrevivência foram geradas pelo método de Kaplan-Meier, com comparação entre grupos realizada com a análise de sobrevivência, SPSS, versão 19.0. Outras análises estatísticas e gráficos foram gerados com o software GraphPad Prism, versão 6.00 (GraphPad Software Inc). O valor de p menor que 0,05 foi considerado como estatisticamente significante.

## Resultados

### Características demográficas e clínicas

O estudo incluiu um total de 337 pacientes hospitalizados com diagnóstico confirmado de COVID-19, incluindo 112 (33,2%) pacientes diagnosticados com hipertensão no momento da internação. A idade média de todos os pacientes foi 58 anos (18-91), e 171 (50,7%) dos pacientes eram homens. As comorbidades subjacentes mais comuns eram diabetes (49, 14,5%), doença cardiovascular (43, 12.8%) e doença no fígado (24, 7,1%). Dos 337 pacientes, 101 (30,0%) foram categorizados como moderados; 186 (55,2%), como graves; e 50 (14,8%) como críticos. Desses 337 pacientes, 297 (87,8%) receberam alta hospitalar e 40 (11,9%) morreram.

Em comparação com pacientes normotensos, os hipertensos eram mais velhos e, na sua maioria, homens. Além disso, os pacientes com hipertensão apresentaram taxas significativamente mais altas de comorbidades, incluindo diabetes, doença cardiovascular, doenças do fígado, doença renal e doença cerebrovascular. Pacientes hipertensos apresentaram a maior porção de casos graves, sendo 69 [61,6%] vs. 117 [52,0%] em pacientes graves e 23 [19,3%] vs. 27 (12,0%) em pacientes críticos. As taxas de mortalidade foram significativamente mais altas dentre os pacientes hipertensos (20 [17,9%] vs. 20 [8,9%]). ( Tabela 1 ).

**Tabela 1:** – Características demográficas e clínicas de pacientes com COVID-19

	No. (%)			

Característica	Total (n=337)	Normotensos (n=225)	Hipertensos (n=112)	Valor de p
Idade média (variação)	58(18-91)	54(18-88)	65(38-91)	<0,0001^a^
Sexo				,0209^b^
Feminino	166(49,3)	121(53,8)	45(40,2)	
Masculino	171(50,7)	104(46,2)	67(59,8)	
Fumante	26(7,7)	18(8,0)	8(7,1)	1,00 ^b^
Início dos sintomas até internação, mediana (IIQ), d	10 (6-13)	9(6-12)	10(7-15)	,1596 ^a^
Hospitalização, mediana (IIQ), d	15(11-23)	15,5(11-24)	15(11-22)	,9117 ^a^
**Comorbidade—N. (%)**				
Doença cardiovascular	43(12,8)	11(4,8)	32(28,6)	<0,0001^b^
Doença cerebrovascular	6(1,7)	0	6(5,4)	0,0012^b^
Diabetes	49(14,5)	15(6,7)	34(30,4)	<0,0001^b^
Bronquite crônica	8(2,4)	4(2,2)	4(3,6)	,4480 ^b^
Asma	1(0,3)	1(0,8)	0(0)	1 ^b^
Malignidade	18(5,3)	9(4,0)	9(8,0)	,2924 ^b^
Doença hepática	24(7,1)	9(4,0)	15(13,4)	,0028 ^b^
Doença renal	17(5,0)	5(2,2)	12(10,7)	,0022 ^b^
Alergias	13(3,9)	11(4,9)	2(1,8)	,2332 ^b^
**Complicação**				
Infecção bacteriana	36(10,7)	23(10,2)	13(11,6)	,7106 ^b^
Acidose metabólica	14(4,2)	6(2,7)	8(7,1)	,0784 ^b^
Insuficiência cardíaca	20(5,7)	10(4,4)	10(8,9)	,1398 ^b^
ARDS	42(12,5)	18(8,0)	24(21,4)	,0007 ^b^
Lesão hepatica aguda	17(5,0)	11(4,9)	6(5,3)	1 ^b^
Lesão renal aguda	19(5,6)	8(3,6)	11(9,8)	,0244 ^b^
DIC	4(1,2)	1(0,4)	3(2,7)	,1089 ^b^
**Tratamentos**				
Tratamento antiviral	276(81,9)	193(85,8)	83(74,1)	0,0107 ^b^
Antibióticos	302(89,6)	200(88,9)	102(91,1)	0,5763 ^b^
Medicamento chinês	186(55,2)	122(54,2)	64(57,1)	0,6430 ^b^
Glicocorticoide	150(44,5)	90(40,0)	60(53,6)	0,0202 ^b^
Imunoglobulina	56(16,6)	36(15,6)	21(18,4)	0,3445 ^b^
**Apoio respiratório**				0,0041 ^b^
Cânula nasal	226(67,1)	158(70,2)	68(60,7)	
Ventilação não-invasiva	26(7,7)	10(4,4)	16(14,3)	
Ventilação invasiva	16(4,7)	9(4,0)	7(6,3)	
**Gravidade da doença**				0,0014 ^b^
Moderada	101(30,0)	81(36,0)	20(17,9)	
Grave	186(55,2)	117(52,0)	69(61,6)	
Crítica	50(14,8)	27(12,0)	23(20,5)	
**Resultados clínicos**				,0202 ^b^
Alta	297(87,8)	205(90,7)	92(82,1)	
Morte	40(11,9)	20(8,9)	20(17,9)	

*ARDS: síndrome de aflição respiratória aguda; CID: coagulação intravascular disseminada; IIQ: intervalo interquartil. a: a diferença estatística (variável numérica) entre grupos de pacientes normotensos e hipertensos foi avaliada com o teste U de Mann-Whitney. b: a diferença estatística (variável categórica) entre grupos de pacientes normotensos e hipertensos foi avaliada com o teste de Chi-quadrado.*

### Resultados laboratoriais na internação

Como demonstrado na Tabela 2 , no estudo geral da população de 337 pacientes, o nível médio de PCR e SAA foi alto, e a contagem de linfócitos, proteínas e albumina decresceu. Porém, os outros indicadores laboratoriais estavam normais, incluindo outras contagens sanguíneas, lipídios e eletrólitos, biomarcadores cardíacos, análise de gases sanguíneos e outros marcadores da função hepática e renal.

**Tabela 2:** – Resultados laboratoriais entre os diferentes grupos

	Mediana (IIQ)			

Característica	Total (n=337)	Normotensos (n=225)	Hipertensos (n=112)	Valor de p^a^
**Contagem das células sanguíneas**				
Contagem de leucócitos, ×10^9^/L (variação normal 3,5-9,5)	4,81(3.81-6.57)	4,65(3,63-5,97)	5,61(4,08-7,82)	,0005
Contagem de neutrófilos, ×10^9^/L (variação normal 1,6-6,3)	3,24(2.25-5.02)	2,96(2,13-4,25)	3,91(2,89-6,78)	<0,0001
Contagem de linfócitos, ×10^9^/L (variação normal 1,1-3,2)	0,89(0.63-1.25)	0,97(0,66-1,33)	0,76(0,58-1,10)	0,0011
Contagem de monócitos, ×10^9^/L (variação normal 0,1-0,6)	0,37(0.26-0.50)	0,36(0,26-0,49)	0,41(0,26-0,54)	0,1051
Contagem de plaquetas, ×10^9^/L (variação normal 125-350)	181(132-232)	181,5(132,8-227,3)	180(130-238)	0,8235
**Lipídios do sangue e eletrólitos**				
Colesterol total, mmol/L, (variação normal 2,8-5,2)	3,53(3.01-4.17)	3,43(2,99-4,13)	3,70(3,06-4,17)	0,1034
Triglicérides, mmol/L, (variação normal 0,56-1,7)	0,93(0.69-1.35)	0,88(0,64-1,31)	1,01(0,77-1,58)	0,0127
HDL, mmol/L, (variação normal 0,9-2,1)	1,1(0.92-1.31)	1,11(0,93-1,31)	1,09(0,90-1,30)	0,6562
LDL, mmol/L, (variação normal 1-3,35)	2,02(1.64-2.48)	1,92(1,63-2,44)	2,1(1,67-2,61)	0,0463
sdLDL, mmol/L, (variação normal 95-538)	121(86-184)	115,0(81-174)	131(93-194)	0,0976
**Soro**				
Potássio, mmol/L (variação normal 3,5-5,3)	3,71(3.38-4.07)	3,72(3,43-4,05)	3,71(3,29-4,17)	0,7970
Cálcio, mmol/L (variação normal 2,11-2,52)	2,16(2.07-2.26)	2,17(2,09-2,27)	2,14(2,05-2,24)	0,0612
**Biomarcadores inflamatórios**				
PCRas, mg/L (variação normal 0-3)	31,70(9.08-65,52)	27,2(6,6-61,3)	44,2(14,55-76,05)	,015
Procalcitonina, ng/mL (variação normal 0-0,1)	0,065(0.04-0,14)	0,0525(0,04-0,12)	0,09 (0,05-0,21)	<0,0001
SAA, mg/L (variação normal 0-10)	93,61(32.24-196,1)	104,7(27,57-223,3)	86,16(38,77-159,6)	,5855
Biomarcadores cardíacos				
TnT, ng/mL (variação normal 0-0,014)	0,009(0.006-0,014)	0,008(0,005-0,013)	0,012(0,008-0,0215)	<0,0001
Creatinina-quinase-MB, ng/mL (variação normal 0-6,22)	1,12(0.68-2.31)	1,00 (0,66-1,93)	1,53(0,93-3,05)	0,0005
Mioglobina, ng/mL (variação normal 7,4-105,7)	47,20(27.80-86,00)	40,9(25,90-67,45)	67,20(30,65-131,7)	0,0004
NT-proBNP, pg/mL (variação normal 0-222)	198,4(55.38-488,7)	124,8(47,75-386,6)	243,8(107,1-809,3)	0,0021
**Análise de gases do sangue**				
PaO2, mm Hg (variação normal 70-107)	85,0(62.3-118.0	93(74-121,5)	77(56,0-110,0)	0,0095
PaO2/FiO2, mm Hg	376,2(229.3-469,0)	390,5(274,5-504,8)	293,1(168.3-419,1)	0,0003
PaCO2, mm Hg (variação normal 35-45)	38(33-44)	39(34-44)	36(32-44)	0,0829
PH (variação normal 7,35-7,45)	7,42(7.40-7.46)	7,42(7,40-7,45)	7,43(7,40-7,46)	0,4852
BE, mmol/L, (variação normal -3-3)	1,3(-0.7-3.0)	1,4(0-3,1)	0,6(-1,9-2,8)	0,0646
**Função hepática e renal**				
Alanina Aminotransferase, IU/L (variação normal 9-50)	25,0(16.0-38.0)	23(15,75-34)	27,0(16,0-47,0)	0,0252
Aspartato Aminotransferase, IU/L (variação normal 15-40)	28,0(19.0-40.0)	26(18,0-37,0)	29(20,0-45,0)	0,0382
Proteína total, g/L (variação normal 65-85)	63,7(60.2-67.3)	64,10(60,3-67,3)	63,5(59,5-67,2)	0,5332
Albumina, g/L (variação normal 40-55)	36,5(33.0-39.3)	37,4(34,1-40,1)	33,7(31,3-38,0)	<0,0001
Globulina, g/L, (variação normal 20-40)	27,0 (25.1-30.4)	26,5(24,5-29,0)	28,6(26,2-32,4)	<0,0001
Bilirrubina total, μmol/L (variação normal 2-23)	7,8(5.6-11.0)	7,2(5,3-9,8)	9,6(6,6-12,5)	0,0001
Bilirrubina direta, μmol/L (variação normal 0-8)	3,0(2.2-4.4)	2,9(2,1-4,0)	3,3(2,4-4,9)	0,0138
Creatinina, μmol/L (variação normal 57-97)	64,0(53.0-75.0)	62(52,3-73,0)	68(54,5-85,8)	0,0144
Nitrogênio ureico, μmol/L (variação normal 3,1-8)	4,24(3.36-5.80)	4,13(3,34-5,37)	4,88(3,51-6,26)	0,0113

*IIQ: interval interquartil; HDL: lipoproteína de alta densidade; LDL: lipoproteína de baixa densidade; sdLDL: lipoproteína de baixa densidade pequena e densa; PCRas: proteína C reativa de alta sensibilidade; TnT: troponina T; NT-proBNP: pró-peptídeo natriurético cerebral N-terminal. a: a diferença estatística (variável numérica) entre os grupos de pacientes normotensos e hipertensos foi avaliada com o Teste U de Mann-Whitney.*

Em comparação a pacientes normotensos, os hipertensos apresentaram contagem significativamente maior de leucócitos e neutrófilos, e contagem menor de linfócitos. A contagem de monócitos e plaquetas desses dois grupos não apresentou diferenças.

Os níveis de colesterol total, lipoproteína de alta densidade (HDL) e lipoproteína de baixa densidade pequena e densa (sdLDL) não apresentaram diferenças entre o grupo de pacientes hipertensos e normotensos, mas pacientes com hipertensão apresentaram níveis mais altos de triglicérides e lipoproteína de baixa densidade (LDL). Os biomarcadores inflamatórios, incluindo PCR de alta sensibilidade, procalcitonina e globulina foram significativamente maiores em pacientes hipertensos.

Vale a pena observar que pacientes hipertensos apresentaram níveis anormais de vários indicadores relacionados à função do coração, fígado, rins e pulmão. Os pacientes hipertensos apresentaram níveis significativamente maiores de biomarcadores de lesão cardíaca, incluindo troponina T, creatinina quinase-banda miocárdica, mioglobina e pró-peptídeo natriurético cerebral N-terminal (NT-proBNP). Além disso, pacientes hipertensos apresentaram disfunções respiratórias mais graves, com pressão parcial de oxigênio (PaO2) mais baixa, e fração inspirada de oxigênio (FiO2). Pacientes hipertensos também apresentaram níveis mais altos de creatinina e nitrogênio ureico. Pacientes hipertensos apresentaram níveis mais altos de alanina aminotransferase, aspartato aminotransferase, bilirrubina total, bilirrubina direta e níveis mais baixos de albumina.

### Tratamento, complicações e resultado clínico

O tempo médio desde o início dos sintomas foi de dez dias (IIQ, 7-15) em pacientes hipertensos, assim como pacientes normotensos ( Tabela 1 ). Não houve diferença significativa no tempo de hospitalização entre ambos os grupos. Durante a hospitalização, pacientes hipertensos desenvolveram complicações mais frequentes relacionadas à síndrome de aflição respiratória aguda e lesão renal aguda em comparação a pacientes normotensos ( Tabela 1 ). Mas não houve diferenças significativas em relação à incidência de insuficiência cardíaca aguda e insuficiência hepática aguda entre os dois grupos.

Um total de 268 pacientes (79,5%) precisou de assistência respiratória, e o uso de cânulas nasais, ventilação não-invasiva e ventilação mecânica invasiva foi necessário para 226 (67,1%), 26 (7,7%), e 16 pacientes (4,7%), respectivamente. A maioria dos pacientes recebeu terapia antiviral (276 [81,9%]) e terapia antibacteriana (302 [89,6%]) durante a internação. A proporção do tratamento com medicamentos chineses, glicocorticoides e imunoglobulina foi de 186 (55,2%), 150 (44,5%) e 56 (16,6%), respectivamente. Além disso, as taxas desses tratamentos não apresentaram diferenças significativas entre os grupos com e sem hipertensão. Porém, vale observar que pacientes hipertensos receberam tratamento com glicocorticoide.

De acordo com os critérios diagnósticos, havia 73 (65,1%) pacientes com hipertensão estágio I; 24 (21,4%) com hipertensão estágio II; e 15 (13,4%) com hipertensão estágio III, respectivamente. Todos os pacientes do estágio III apresentaram os tipos graves ou críticos de COVID-19. Mais da metade dos pacientes do estágio III faleceram ( Tabela 3 ).

**Tabela 3:** – A associação entre o estágio de hipertensão e a gravidade da doença em pacientes hipertensos com COVID-19

		Estágio da hipertensão			

	Total (n=112)	I (n=73)	II (n=24)	III (n=15)	Valor de p
**Gravidade da doença**					0,0003^a^
Moderado	20(17,8)	12(16,4)	8(33,3)	0(0)	
Grave	69(61,6)	50(68,5)	13((54,2)	6(40)	
Crítico	23(19,3)	11(15,1)	3(12,5)	9(60)	
**Resultados clínicos**					0,0006^b^
Alta	92(82,1)	64(87,7)	21(87,5)	7(467)	
Morte	20(17,9)	9(12,3)	3(12,5)	8(53,3)	

*a: Teste de Kruskal-Wallis foi usado para analisar a relação entre a gravidade da doença e os estágios da hipertensão. b: R X C O teste de Qui-quadrado foi usado para analisar a relação entre resultados clínicos e estágios de hipertensão.*

Oitenta e quatro (75%) pacientes hipertensos receberam tratamento anti-hipertensivo durante a internação. Entre eles, 20 pacientes (17,8%) usaram inibidores da enzima de conversão da angiotensina (IECAs)/ bloqueadores de receptores da angiotensina (BRAs), e 64 (57,1%) receberam outros medicamentos anti-hipertensivos. A gravidade da doença e os resultados clínicos entre os grupos IECA/BRA e o não-IECA/BRA não apresentaram diferenças significativas ( Tabela 4 ).

**Tabela 4:** – Associação entre o uso de IECA/BRA e a gravidade da doença em pacientes hipertensos com hipertensão

		Tratamento anti-hipertensivo			

	Total (n=112)	Tratamento com IECA/BRA (n=20)	Outros medicamentos hipotensivos (n=64)	Nenhum tratamento hipotensivo (n=28)	Valor de p
**Gravidade da doença**					0,3487^a^
Moderada	20(17,8)	3(15)	11(17,2)	6(21,4)	
Grave	69(61,6)	13(65)	36(56,3)	20(71,4)	
Crítica	23(19,3)	4(20)	17(26,6)	2(7,1)	
**Resultado clínico**					1,0000 ^a^
Alta	92(82,1)	16(80)	49(76,6)	27(96,4)	
Morte	20(17,9)	4(20)	15(23,4)	1(3,6)	

*IECA: inibidores da enzima de conversão da angiotensina; BRA: bloqueadores de receptores da angiotensina. a: R X C o teste de Qui-quadrado foi usado para analisar a diferença entre os grupos.*

### Mudanças dinâmicas nos níveis durante a internação

**Figura 1:**
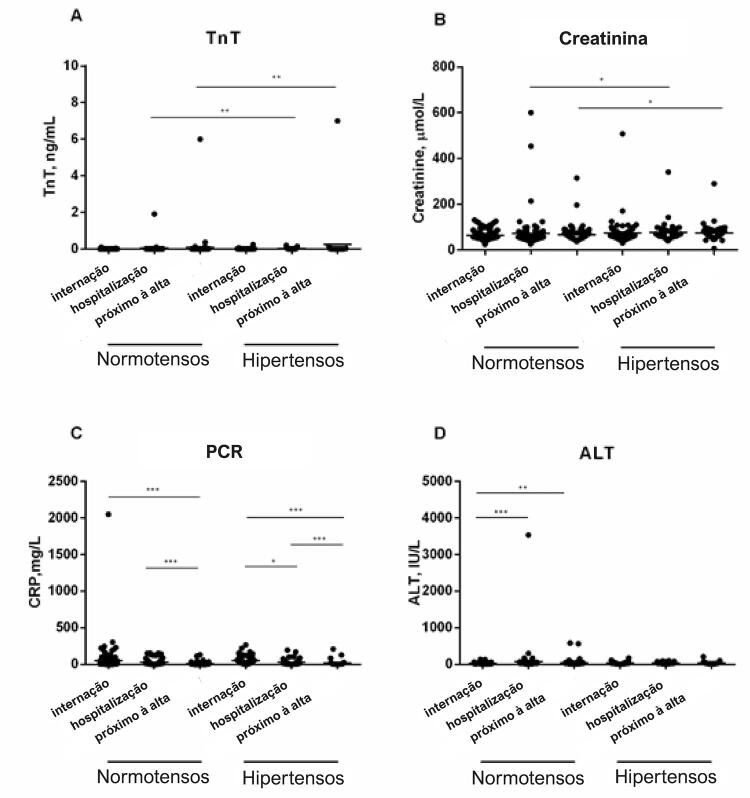
– Mudança dinâmica nos níveis de TnT, Creatinina, PCR e ALT durante a hospitalização. A.TnT; B. Creatinina; C.PCR; D.ALT. Os dados foram demonstrados pela mediana e IIQ. O teste U de Mann-Whitney foi utilizado. (*p < 0,05, **p < 0,01, ***p < 0,001).

Já que os pacientes com hipertensão apresentaram níveis mais altos de PCR, TnT, creatinina e ALT, em comparação a pacientes normotensos, analisamos a mudança dinâmica desses quatro marcadores laboratoriais durante a internação entre os pacientes sobreviventes ( Figura 1 ). Como demonstrado na Figura 1A , o nível de TnT em pacientes hipertensos aumentou significativamente durante o progresso da internação em comparação a pacientes normotensos (mediana [IIQ], 0,011 [0,008-0,021] vs. 0,008 [0,005-0,014], p=0,0027 na internação e 0,012 [0,008-0,165] vs. 0,006 [0,005-0,012], p=0,0077 próximo à alta). Não foi observada a ascensão dinâmica nos níveis de TnT em pacientes normotensos. Da mesma forma, o nível de creatinina em pacientes hipertensos aumentou significativamente durante a internação em comparação aos normotensos (mediana [IIQ], 69,0 [59,5-85,5] vs. 63,0 [51,3-75,8], p=0,0227 na internação e 70,0 [59,0-84,0] vs. 64,0 [51,0-75], p=0,0220) próximo à alta ( Figura 1B ).

Ambos os grupos de pacientes tinham níveis altos de PCR durante a internação. A PCR de pacientes normotensos foi controlada a níveis normais (mediana [IIQ], 2,75[1,0-8,075]), sem diferenças significativas em relação ao grupo dos hipertensos (mediana [IIQ], 3,8[2,2-10,00]) próximo à alta ( Figura 1C ). Da mesma forma, não havia diferenças significativas no nível de ALT entre esses dois grupos no momento próximo à alta ( Figura 1D ).

### A hipertensão aumenta a taxa de mortalidade em pacientes com COVID-19

A relação entre hipertensão e morte foi um dos focos do nosso estudo. Verificamos que as taxas de mortalidade em grupos com hipertensão eram maiores do que grupos sem hipertensão. Enquanto isso, a hipertensão esteve associada a quase 2,2 mais chances de morrer devido à COVID-19 (OR: 2,093 [IC95%: 1,094-4,006], p=0,024), de acordo com o teste de Qui-quadrado.

**Figura 2:**
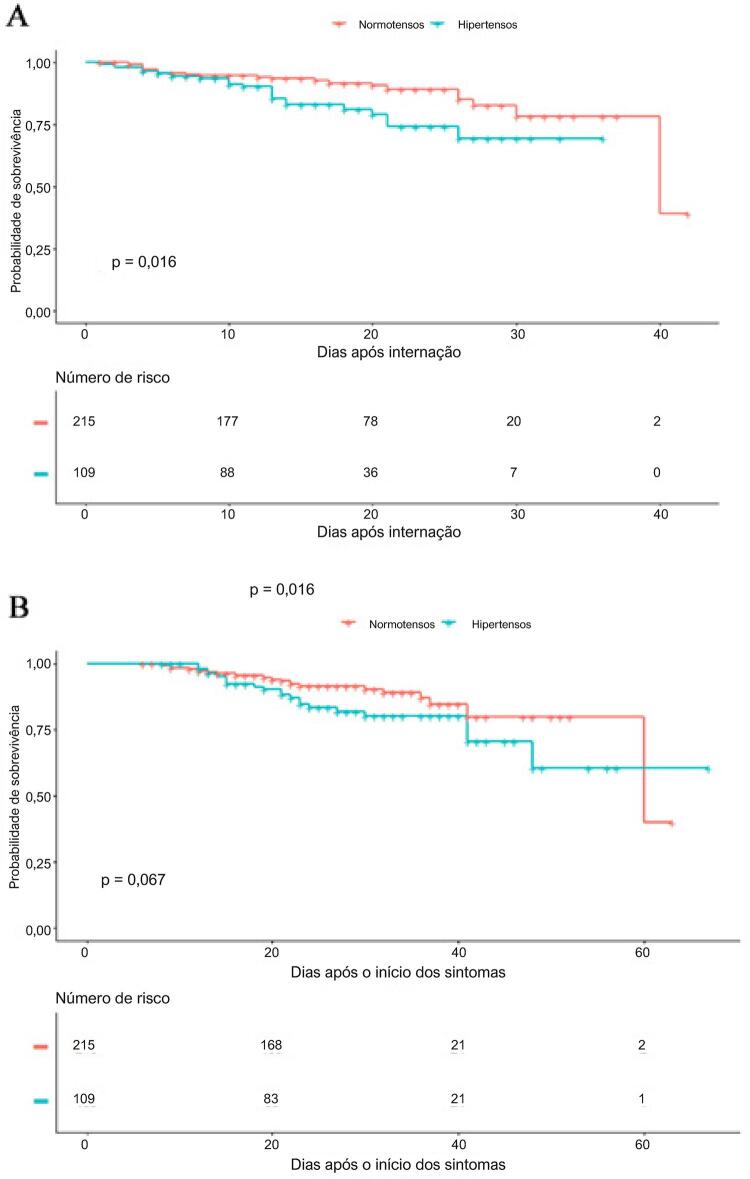
– *Teste de Kaplan–Meier para calcular a probabilidade de sobrevivência em pacientes hospitalizados com COVID-19. A. Curvas de sobrevivência de Kaplan-Meier para mortalidade durante o período após internação. B. Curvas de sobrevivência de Kaplan-Meier para mortalidade durante o período após o início dos sintomas.*

Realizamos uma análise de curva de sobrevivência utilizando o método de Kaplan-Meier. Pacientes hipertensos e normotensos apresentaram curvas de sobrevivência diferentes desde a internação até o acompanhamento (média=31.664, SED=1.424; média=34,79, SED=0,882; p=0,0155), como demonstrado na Figura 2A . Considerando a duração da doença no momento da internação, também observamos que a curva de sobrevivência dos pacientes com e sem hipertensão não apresentou diferenças significativas durante o tempo entre o início dos sintomas e o acompanhamento (média=51.984, SE=2.703; média=55.625, SE=2.139; p>0,05, Figura 2B ).

## Discussão

O mundo atualmente está sofrendo com uma doença infecciosa – COVID-19, que tinha 30.949.804 casos confirmados e 959.116 mortes até 21 de setembro de 2020.^17^ Vários estudos demonstraram que a hipertensão foi a comorbidade mais relacionada a pacientes com COVID-19.^1 , 5^ Na amostra deste estudo, detalhamos as características clínicas e os fatores de risco associados aos resultados clínicos da COVID-19 em pacientes hipertensos e normotensos. A taxa geral de mortalidade na China foi de 5,5% (4.642 mortes dos 84.393 casos confirmados até 3 de maio de 2020).^17^ Em nosso estudo, a prevalência de hipertensão em pacientes com COVID-19 foi de 33,2%, o que está de acordo com estudos anteriores que reportaram a proporção de pacientes com COVID-19 com hipertensão variando entre 19,4 e 32,6%.^5 , 13 , 18^ A mortalidade hospitalar em pacientes com hipertensão é muito maior do que em pacientes normotensos (17,9% vs. 8,9%, p=0,0202), similar a estudos anteriores.

Como se sabe, a ECA2, como uma enzima do sistema renina-angiotensina (SRA), é receptora para o SARS-CoV-2, e essencial para a entrada viral.^19^ A ECA2 não só se expressa na célula epitelial alveolar tipo 2 nos pulmões, mas também no túbulo renal, nos rins; cardiomiócitos, no coração; epitélio do intestino delgado, no trato intestinal; células epiteliais dos ductos biliares e células Leydig, nos testículos.^20^ Assim, pacientes com COVID-19 apresentaram múltiplas manifestações extrapulmonares e possíveis complicações. Na nossa amostra, pacientes hipertensos com COVID-19 tinham mais comorbidades, como diabetes, doença cardiovascular, doenças no fígado, doença renal e doença cerebrovascular. Assim, pacientes hipertensos com COVID-19 apresentaram níveis anormais de vários indicadores relacionados à função cardíaca, hepática, renal e pulmonar no momento da internação. Além disso, pacientes com COVID-19 com hipertensão tinham níveis mais altos de triglicérides e bilirrubina direta. Também resumimos outros parâmetros laboratoriais que podem estar associados à piora da COVID-19 em pacientes hipertensos. Vale lembrar que pacientes hipertensos apresentaram contagem de leucócitos e neutrófilos significativamente mais alta, e contagem de linfócitos mais baixa, o que indica que o nível de linfopenia é maior em pacientes hipertensos com COVID-19, que possuem contagem mais baixa de linfócitos e contagem mais alta de leucócitos e relação de neutrófilos/linfócitos (RNL),^21^ com número extremamente menor de subpopulações de linfócitos e maior nível de citocinas pró-inflamatórias, incluindo IL-2, IL-6 e IL-10.^22^ É importante determinar se pacientes com COVID-19 com hipertensão também apresentaram desregulação grave da resposta imunológica em comparação a pacientes normotensos, mas a vigilância da linfopenia pode ajudar no tratamento de pacientes hipertensos com COVID-19.

Também analisamos as mudanças dinâmicas em quatro biomarcadores durante a internação, e verificamos que ALT (lesão no fígado) e PCR (biomarcadores inflamatórios) não mudaram significativamente entre os grupos com e sem hipertensão. Embora o grupo com hipertensão tenha mostrado uma relação um pouco maior de infecções bacterianas, sem significância estatística, descobrimos que a infecção bacteriana levou a mais chances de mortalidade (OR: 5.867, IC95%: 2.537-13.568, p<0,001). Porém, a hipertensão ainda era um fator de risco independentemente relacionado à mortalidade após o ajuste do efeito da infecção bacteriana (OR: 2.029, IC95%: 1.035-3.976, p<0,05), e o médico deve prestar atenção à infecção bacteriana secundária no grupo com hipertensão em relação aos níveis mais altos de PCR. Porém, os níveis de TnT e creatinina no grupo com hipertensão estavam significativamente mais altos do que no grupo sem hipertensão durante a internação e próximo à alta, o que implica que um manejo clínico mais agressivo em relação à lesão cardíaca e renal pode ser necessário para pacientes hipertensos com COVID-19. Observou-se que os componentes do SRA podem ter papel patogênico na COVID-19, já que a ECA2 age diretamente na hipertensão e na transmissão da SARS-CoV-2.^4^ O equilíbrio da via SRA pode estabelecer a ocorrência de lesão tecidual, principalmente no coração e nos rins.^20^ Nossos dados reforçaram a influência da hipertensão na gravidade da COVID-19, principalmente na lesão cardíaca e renal.

Não é surpreendente que pacientes hipertensos com COVID-19 estejam vivenciando a maior frequência, formas graves e mais complicações da COVID-19. Nossas análises demonstraram que o estágio da hipertensão esteve associado à gravidade da doença e ao resultado clínico em pacientes hipertensos com COVID-19. Porém, os mecanismos que baseiam a relação entre a hipertensão e a COVID-19 não são bem compreendidos. Como a ECA2 age como receptora para que a SARS-CoV-2 entre nas células, há preocupações crescentes sobre o uso clínico do IECA/BRA, ou seja, se esses medicamentos podem ou não aumentar a suscetibilidade a uma infecção de SARS-CoV-2.^23^ Nossos dados demonstraram que o IECA/BRA não aumentaria a gravidade da doença ou risco de mortalidade em pacientes hipertensos com COVID-19. Recentemente, um estudo multicêntrico incluindo 1.128 pacientes hipertensos com COVID-19 mostrou que o uso hospitalar do IECA/BRA esteve associado a taxas mais baixas de mortalidade em comparação aos pacientes que não utilizaram IECA/BRA.^14^ Combinados com nossos resultados, esses achados sugerem que o uso contínuo de IECA/BRA durante a internação deve ser mantido para controlar a pressão arterial para o benefício do paciente, já que pacientes com COVID-19 usando IECA/BRA não apresentaram riscos maiores para resultados adversos.

Porém, este estudo tem algumas limitações. Primeiro, os pacientes sem grandes complicações foram destinados a centros temporários de tratamento (hospitais móveis de campanha), devido aos recursos médicos limitados, e todos os pacientes do estudo tinham casos relativamente graves de COVID-19. Em segundo lugar, os dados do acompanhamento médico estavam incompletos, já que casos críticos foram transferidos para a UTI ou para um hospital superior. Essas medidas foram conduzidas de acordo com estratégias nacionais para prevenção e controle da epidemia, considerando a emergência do surto da COVID-19, que tem grande importância para mitigar a disseminação do vírus. Em terceiro lugar, somente 20 pacientes receberam tratamento com IECA/BRA, o que pode ter limitado a determinação do uso potencial do IECA/BRA no tratamento da COVID-19. Mais investigações clínicas são necessárias.

## Conclusão

Este estudo sugeriu que a hipertensão tem associação significativa com a gravidade e a mortalidade da COVID-19. Pacientes hipertensos com COVID-19 apresentaram severas manifestações e complicações em outros órgãos, principalmente lesões no miocárdio e nos rins, o que implica que tratamentos agressivos devam ser considerados para pacientes hipertensos diagnosticados com COVID-19. A observação de longo prazo e um estudo prospectivo sobre a efetividade dos tratamentos específicos para a COVID-19 em pacientes hipertensos são necessários.
