# Supplementary material for: Associação da Hipertensão com a Gravidade e a Mortalidade de Pacientes Hospitalizados com COVID-19 em Wuhan, China: Estudo Unicêntrico e Retrospectivo
Source: Arq Bras Cardiol. 2021 Jul 7;117(5):911–21. [Article in Portuguese] doi: 10.36660/abc.20200733 (PMC8682108; doi:10.36660/abc.20200733)
Supplement: Translation

# Translation: Association of Hypertension with Severity and Mortality in Hospitalized Patients with COVID-19 in Wuhan, China: A Single-centered, Retrospective Study

**Authors:** You-ping Deng, Wen Xie, Tao Liu, Shou-yi Wang, Mei-rong Wang, Yu-xing Zan, Xiao-bo Meng, Yu-qing Deng, Hai-rong Xiong, Xue-dong Fu

**Affiliations:** 1 China Department of Pediatrics, Zhongnan Hospital of Wuhan University, Wuhan - China; 2 China Department of Clinical Laboratory, Zhongnan Hospital of Wuhan University , Wuhan - China; 3 China Department of Urology, Zhongnan Hospital of Wuhan University, Wuhan - China; 4 China State Key Laboratory of Virology/ Institute of Medical Virology, School of Basic Medical Sciences, Wuhan University, Wuhan - China

**Keywords:** COVID-19/complications, Betacoronavirus, Severe Acute Respiratoty Syndrome, Hypertension, Comorbidities, Risk Factors

## Abstract

**Background:**

Coronavirus disease 2019 (COVID-19), caused by the severe acute respiratory syndrome coronavirus 2 (SARS-CoV-2), has spread worldwide.

**Objective:**

To investigate the association between hypertension and severity/mortality in hospitalized patients with COVID-19 in Wuhan, China.

**Methods:**

A total of 337 patients diagnosed with COVID-19 at the Seventh Hospital of Wuhan City, from January 20 to February 25, 2020, were enrolled and analyzed in a retrospective, single-center case study. The significance level adopted in the statistical analysis was 0.05.

**Results:**

Of the 337 patients with confirmed diagnosis of COVID-19, 297 (87.8%) were discharged from the hospital and 40 patients (22.9%) died. The median age was 58 years (range, 18-91 years). There were 112 (33.2%) patients diagnosed with hypertension at admission (median age, 65.0 years [range, 38-91 years]; 67 [59.8%, 95%CI: 50.6%-69.0%] men, p=0.0209). Patients with hypertension presented a significantly higher portion of severe cases (69 [61.6%, 95%CI:52.5%-70.8%] vs. 117 [52.0%, 95%CI: 45.4%-58.6%] in severe patients and 23 [19.3%, 95%CI:12.9%-28.1%] vs. 27 [12.0%, 95%CI: 7.7%-16.3%] in critical patients, p=0.0014) and higher mortality rates (20 [17.9%, 95%CI: 10.7%-25.1%] vs. 20 [8.9%, 95%CI: 5.1%-12.6%, p=0.0202). Moreover, hypertensive patients presented abnormal levels of multiple indicators, such as lymphopenia, inflammation, heart, liver, kidney, and lung function at admission. The hypertension group still displayed higher levels of TnT and creatinine at approaching discharge.

**Conclusion:**

Hypertension is strongly associated with severity or mortality of COVID-19. Aggressive treatment may be considered for COVID-19 patients with hypertension, especially regarding cardiac and kidney injury.

## Introduction

The novel Coronavirus disease 2019 (COVID-19), caused by the severe acute respiratory syndrome coronavirus (SARS-CoV2), emerged in Wuhan in December, 2019, and has spread worldwide, which resulted in great concern to global public health and economics.^1^ SARS-CoV-2 was identified as the pathogen of COVID-19 in January 2020, and belongs to a unique clade of the subgenus sarbecovirus, Orthocoronavirinae subfamily.^2^ This novel coronavirus is an enveloped, single stranded, positive-sense RNA virus, and recognizes angiotensin-converting enzyme-2 (ACE2) as the functional receptor for host cell entry. ACE2 is a member of the angiotensin-converting enzyme (ACE) family and plays an important role in human physiological functions, especially in blood pressure regulation.^3 , 4^ Emerging data reported the general clinical features and epidemiological characteristics of COVID-19 patients, among which several reports demonstrated that cardiac injury is associated with higher risk of mortality in patients with COVID-19.^5 - 7^

There is a high prevalence of hypertension worldwide, especially in China. Overall, hypertension was present in 23.2% of the adult Chinese population from 2012 to 2015.^8^ Hypertension is a major risk factor for cardiovascular disease, the leading cause of death in China.^9 , 10^ With urbanization, rising incomes and an aging population, China’s burden of hypertension and cardiovascular disease is increasing.^11 , 12^ Accumulated evidence suggested that hypertension may be related to a growing factor of in-hospital fatality due to COVID-19.^13 , 14^ Therefore, we initiated this retrospective study to analyze data from a single center in Wuhan, China, and examine the association between hypertension and COVID-19. We also monitored the dynamic changes of important biomarkers among the hospitalized patients, which may bring recommendations for the clinical management of hypertensive patients with COVID-19.

## Methods

### Patients’ enrollment

The institutional Ethics board of Zhongnan Hospital, Wuhan University, approved this project (No.2020056K). No.7 Hospital of Wuhan was one of the first COVID-19 designated hospitals, and has been consigned to the Zhongnan Hospital of Wuhan University since January 2020. A total of 337 patients with confirmed diagnosis of COVID-19 hospitalized in four inpatient wards of No.7 Hospital of Wuhan were enrolled in this study, which was conducted from January 20 to February 25, 2020. All patients were diagnosed with COVID-19 and classified in distinct clinical types, according to the diagnostic and treatment guidelines of COVID-19 from the Chinese National Health Commission (version 3-7).^15^ Since patients without major complications were assigned to the mobile cabin hospital due to the hierarchical medical system during the early stage of the epidemic, all the patients involved in this study had moderate (101), severe (186), and critical (50) COVID-19. The critical cases were further transferred to the ICU. The throat swab samples were collected and applied for laboratory detection.

### Data collection

The medical records, including basic information (age, gender, comorbidities etc.), clinical characteristics, laboratory findings and radiological examinations, as well as treatment and outcomes of each patient with positive SARS-CoV-2 results, were collected. The date of disease onset was specified as the day when any symptom was observed. Clinical outcomes were evaluated and recorded at the time of discharge or transfer to the assigned intensive care hospital. Laboratory confirmation of SARS-CoV-2 was primarily performed in the clinical laboratory at the Zhongnan Hospital of Wuhan University, and partially in the clinical laboratory at No.7 Hospital of Wuhan after the detection system was locally established since late February. SARS-CoV-2 was verified by real-time RT-PCR using the protocol as previously described.^16^ The viral nucleic acid detection from throat-swab specimen was performed during the therapeutic process. Besides, the patient samples were also detected for other pathogen infections, such as influenza virus, parainfluenza, Coxsackie virus, adenovirus, echovirus, respiratory syncytial virus, cytomegalovirus etc. All the patients underwent chest computed tomography (CT-scan) or X-ray. Follow-up radiological examination and negative SARS-CoV-2 test results were considered as criteria for cure and hospital discharge.

Clinical manifestations were summarized, including fever, cough, expectoration, myalgia, fatigue, headache, heart palpitations, diarrhea, dyspnea etc. Laboratory examinations were conducted at admission and as the disease progressed, such as routine blood tests, blood biochemistry, blood gas level, blood electrolytes, coagulation function, procalcitonin (PCT), C-reactive protein (CRP), serum amyloid A (SAA), serum creatine kinase and myocardial enzyme spectrum. Medical treatments were recorded, as most patients received the antiviral treatment or a Chinese patent medicine. Patients also received corticosteroid, gamma globulin, probiotics, or respiratory support when necessary.

### Statistical analysis

Categorical data were presented as frequencies and percentage rates, and continuous data were described using median and interquartile range (IQR) values. The continuous variables were tested for Gaussian distribution using the D’Agostino-Pearson test for normality and further analyzed by the Mann-Whitney test, when appropriate. The frequencies of categorical variables were compared using the chi-square test, Fisher’s exact test, and Kruskal-Wallis when appropriate. Survival curves were generated by the Kaplan-Meier method, with comparisons between groups performed with the log-rank test, SPSS version 19.0. Other statistical analyses and graphs were generated and plotted using the GraphPad Prism software, version 6.00 (GraphPad Software Inc). *P* value lower than 0.05 was considered as statistically significant.

## Results

### Demographics and clinical characteristics

The study enrolled a total of 337 patients hospitalized with confirmed diagnosis of COVID-19, including 112 (33.2%) patients diagnosed as hypertensive at admission. The median age for all patients was 58 years (18-91), and 171 (50.7%) patients were male. The most common underlying comorbidities were diabetes (49, 14.5%), cardiovascular disease (43, 12.8%), and liver disease (24, 7.1%). Of the 337 patients, 101 (30.0%) were categorized as moderate patients; 186 (55.2%) as severe patients; and 50 (14.8%) as critical patients. Of these 337 patients, 297 (87.8%) were discharged from the hospital and 40 (11.9%) patients died.

Compared with non-hypertensive patients, hypertensive patients were older and most were male. Moreover, patients with hypertension presented significantly higher rates of comorbidities, including diabetes, cardiovascular disease, liver disease, kidney disease and cerebrovascular disease. Patients with hypertension presented a significantly higher portion of severe cases, as 69 [61.6%] vs. 117 [52.0%] in severe patients and 23 [19.3%] vs. 27 [12.0%] in critical patients. Mortality rates were significantly higher among patients with hypertension (20 [17.9%] vs. 20 [8.9%]). ( Table 1 ).

**Table 1:** – Demographics and clinical characteristics of patients with COVID-19

	No. (%)			

Characteristic	Total (n=337)	Normotension (n=225)	Hypertension (n=112)	p-value
Mean age (range)	58(18-91)	54(18-88)	65(38-91)	<0.0001^a^
Sex				.0209^b^
Female	166(49.3)	121(53.8)	45(40.2)	
Male	171(50.7)	104(46.2)	67(59.8)	
Smoking	26(7.7)	18(8.0)	8(7.1)	1.00^b^
Onset of symptoms to hospital admission, median (IQR), d	10 (6-13)	9(6-12)	10(7-15)	.1596^a^
Hospitalization, median (IQR), d	15(11-23)	15.5(11-24)	15(11-22)	.9117^a^
**Comorbidity—No. (%)**				
Cardiovascular disease	43(12.8)	11(4.8)	32(28.6)	<0.0001^b^
Cerebrovascular disease	6(1.7)	0	6(5.4)	0.0012^b^
Diabetes	49(14.5)	15(6.7)	34(30.4)	<0.0001^b^
Chronic bronchitis	8(2.4)	4(2.2)	4(3.6)	.4480^b^
Asthma	1(0.3)	1(0.8)	0(0)	1^b^
Malignancy	18(5.3)	9(4.0)	9(8.0)	.2924^b^
Liver disease	24(7.1)	9(4.0)	15(13.4)	.0028^b^
Kidney disease	17(5.0)	5(2.2)	12(10.7)	.0022^b^
Allergic physique	13(3.9)	11(4.9)	2(1.8)	.2332^b^
**Complication**				
Bacterial infection	36(10.7)	23(10.2)	13(11.6)	.7106^b^
Metabolic acidosis	14(4.2)	6(2.7)	8(7.1)	.0784^b^
Heart failure	20(5.7)	10(4.4)	10(8.9)	.1398^b^
ARDS	42(12.5)	18(8.0)	24(21.4)	.0007^b^
Acute liver injury	17(5.0)	11(4.9)	6(5.3)	1^b^
Acute kidney injury	19(5.6)	8(3.6)	11(9.8)	.0244^b^
DIC	4(1.2)	1(0.4)	3(2.7)	.1089^b^
**Treatments**				
Antiviral treatment	276(81.9)	193(85.8)	83(74.1)	0.0107^b^
Antibiotics	302(89.6)	200(88.9)	102(91.1)	0.5763^b^
Chinese Medicine	186(55.2)	122(54.2)	64(57.1)	0.6430^b^
Glucocorticoid	150(44.5)	90(40.0)	60(53.6)	0.0202^b^
Immune globulin	56(16.6)	36(15.6)	21(18.4)	0.3445^b^
**Respiratory support**				**0.0041** ^**b**^
Nasal cannula	226(67.1)	158(70.2)	68(60.7)	
Non-invasive ventilation	26(7.7)	10(4.4)	16(14.3)	
Invasive ventilation	16(4.7)	9(4.0)	7(6.3)	
**Disease severity**				**0.0014** ^**b**^
Moderate	101(30.0)	81(36.0)	20(17.9)	
Severe	186(55.2)	117(52.0)	69(61.6)	
Critical	50(14.8)	27(12.0)	23(20.5)	
**Clinical outcomes**				**.0202** ^**b**^
Discharge	297(87.8)	205(90.7)	92(82.1)	
Death	40(11.9)	20(8.9)	20(17.9)	

*ARDS: acute respiratory distress syndrome; DIC: Disseminated intravascular coagulation; IQR: interquartile range. a: statistical difference (numerical variable) between normotension and hypertension groups were evaluated by the Mann-Whitney U test. b: statistical difference (categorical variable) between normotension and hypertension groups were evaluated by the Chi-square test.*

### Laboratory findings at Admission

As shown in Table 2 , in the overall study population of 337 patients, the median level of CRP and SAA was elevated, and lymphocyte count, total protein and albumin were decreased. However, the other laboratory indicators were within the normal range, including other blood cell counts, blood lipids and electrolytes, cardiac biomarkers, blood gas analysis and other liver and renal function biomarkers.

**Table 2:** – Laboratory results among different groups

	Median (IQR)			

Characteristic	Total (n=337)	Normotension (n=225)	Hypertension (n=112)	p-value^a^
**Blood cell count**				
White blood cell count, ×10^9^/L (normal range 3.5-9.5)	4.81(3.81-6.57)	4.65(3.63-5.97)	5.61(4.08-7.82)	.0005
Neutrophil count, ×10^9^/L (normal range 1.6-6.3)	3.24(2.25-5.02)	2.96(2.13-4.25)	3.91(2.89-6.78)	<0.0001
Lymphocyte count, ×10^9^/L (normal range 1.1-3.2)	0.89(0.63-1.25)	0.97(0.66-1.33)	0.76(0.58-1.10)	0.0011
Monocyte count, ×10^9^/L (normal range 0.1-0.6)	0.37(0.26-0.50)	0.36(0.26-0.49)	0.41(0.26-0.54)	0.1051
Platelet count, ×10^9^/L (normal range 125-350)	181(132-232)	181.5(132.8-227.3)	180(130-238)	0.8235
**Blood lipids and electrolytes**				
Total cholesterol, mmol/L, (normal range 2.8-5.2)	3.53(3.01-4.17)	3.43(2.99-4.13)	3.70(3.06-4.17)	0.1034
Triglyceride, mmol/L, (normal range 0.56-1.7)	0.93(0.69-1.35)	0.88(0.64-1.31)	1.01(0.77-1.58)	0.0127
HDL, mmol/L, (normal range 0.9-2.1)	1.1(0.92-1.31)	1.11(0.93-1.31)	1.09(0.90-1.30)	0.6562
LDL, mmol/L, (normal range 1-3.35)	2.02(1.64-2.48)	1.92(1.63-2.44)	2.1(1.67-2.61)	0.0463
sdLDL, mmol/L, (normal range 95-538)	121(86-184)	115.0(81-174)	131(93-194)	0.0976
**Serum**				
Potassium, mmol/L (normal range 3.5-5.3)	3.71(3.38-4.07)	3.72(3.43-4.05)	3.71(3.29-4.17)	0.7970
Calcium, mmol/L (normal range 2.11-2.52)	2.16(2.07-2.26)	2.17(2.09-2.27)	2.14(2.05-2.24)	0.0612
**Inflammatory biomarkers**				
hsCRP, mg/L (normal range 0-3)	31.70(9.08-65.52)	27.2(6.6-61.3)	44.2(14.55-76.05)	.015
Procalcitonin, ng/mL (normal range 0-0.1)	0.065(0.04-0.14)	0.0525(0.04-0.12)	0.09 (0.05-0.21)	<0.0001
SAA, mg/L (normal range 0-10)	93.61(32.24-196.1)	104.7(27.57-223.3)	86.16(38.77-159.6)	.5855
**Cardiac biomarkers**				
TnT, ng/mL (normal range 0-0.014)	0.009(0.006-0.014)	0.008(0.005-0.013)	0.012(0.008-0.0215)	<0.0001
Creatine kinase-MB, ng/mL (normal range 0-6.22)	1.12(0.68-2.31)	1.00 (0.66-1.93)	1.53(0.93-3.05)	0.0005
Myoglobulin, ng/mL (normal range 7.4-105.7)	47.20(27.80-86.00)	40.9(25.90-67.45)	67.20(30.65-131.7)	0.0004
NT-proBNP, pg/mL (normal range 0-222)	198.4(55.38-488.7)	124.8(47.75-386.6)	243.8(107.1-809.3)	0.0021
**Blood gas analysis**				
PaO2, mm Hg (normal range 70-107)	85.0(62.3-118.0	93(74-121.5)	77(56.0-110.0)	0.0095
PaO2/FiO2, mm Hg	376.2(229.3-469.0)	390.5(274.5-504.8)	293.1(168.3-419.1)	0.0003
PaCO2, mm Hg (normal range 35-45)	38(33-44)	39(34-44)	36(32-44)	0.0829
PH (normal range 7.35-7.45)	7.42(7.40-7.46)	7.42(7.40-7.45)	7.43(7.40-7.46)	0.4852
BE, mmol/L, (normal range -3-3)	1.3(-0.7-3.0)	1.4(0-3.1)	0.6(-1.9-2.8)	0.0646
**Liver and renal function**				
Alanine Aminotransferase, IU/L (normal range 9-50)	25.0(16.0-38.0)	23(15.75-34)	27.0(16.0-47.0)	0.0252
Aspartate Aminotransferase, IU/L (normal range 15-40)	28.0(19.0-40.0)	26(18.0-37.0)	29(20.0-45.0)	0.0382
Total Protein, g/L (normal range 65-85)	63.7(60.2-67.3)	64.10(60.3-67.3)	63.5(59.5-67.2)	0.5332
Albumin, g/L (normal range 40-55)	36.5(33.0-39.3)	37.4(34.1-40.1)	33.7(31.3-38.0)	<0.0001
Globulin, g/L, (normal range 20-40)	27.0 (25.1-30.4)	26.5(24.5-29.0)	28.6(26.2-32.4)	<0.0001
Total bilirubin, μmol/L (normal range 2-23)	7.8(5.6-11.0)	7.2(5.3-9.8)	9.6(6.6-12.5)	0.0001
Direct bilirubin, μmol/L (normal range 0-8)	3.0(2.2-4.4)	2.9(2.1-4.0)	3.3(2.4-4.9)	0.0138
Creatinine, μmol/L (normal range 57-97)	64.0(53.0-75.0)	62(52.3-73.0)	68(54.5-85.8)	0.0144
Urea nitrogen, μmol/L (normal range 3.1-8)	4.24(3.36-5.80)	4.13(3.34-5.37)	4.88(3.51-6.26)	0.0113

*IQR: interquartile range; HDL: high-density lipoprotein; LDL: low-density lipoprotein; sdLDL: small dense Low-Density Lipoprotein; hsCRP: high-sensitivity C-reactive protein; TnT: troponin T; NT-proBNP: N-terminal pro–brain natriuretic peptide. a: statistical difference (numerical variable) between normotension and hypertension groups were evaluated by the Mann-Whitney U test.*

Compared with non-hypertensive patients, hypertensive patients presented with significantly higher white blood cell and neutrophil count, and lower lymphocyte count. The monocyte and platelet counts of these two groups were similar.

Total cholesterol, high-density lipoprotein (HDL), and small dense Low-Density Lipoprotein (sdLDL) levels did not differ between hypertensive and non-hypertensive patients, but hypertensive patients had higher levels of triglyceride and low-density lipoprotein (LDL). The inflammatory biomarkers, including highly sensitive CRP, procalcitonin, and globulin were significantly higher in hypertensive patients.

It is worth noting that hypertensive patients presented abnormal levels of multiple indicators concerning the heart, liver, kidney, and lung function. Hypertensive patients presented significantly higher levels of cardiac injury biomarkers, including troponin T, creatine kinase-myocardial band test, myoglobin and N-terminal pro-brain natriuretic peptide (NT-proBNP). Moreover, hypertensive patients showed more severe respiratory dysfunction, with lower partial pressure of oxygen (PaO2), and PaO2/fraction of inspired oxygen (FiO2). Furthermore, hypertensive patients also had higher levels of creatinine and urea nitrogen. Hypertensive patients presented higher levels of alanine aminotransferase, aspartate aminotransferase, total bilirubin, direct bilirubin, and lower levels of albumin.

### Treatment, complications, and clinical outcome

The median time from symptom onset to admission was ten days (IQR, 7-15) in hypertensive patients, and similar with non-hypertensive patients ( Table 1 ). There was no significant difference in hospitalization time between both groups. During hospitalization, hypertensive patients developed more frequent complications regarding acute respiratory distress syndrome, and acute renal injury when compared with non-hypertensive patients ( Table 1 ). But there were no significant differences regarding the incidence of acute heart failure and acute liver injury between these two groups.

A total of 268 patients (79.5%) underwent respiratory support, and the use of nasal cannula, non-invasive ventilation and invasive mechanical ventilation was necessary for 226 (67.1%), 26 (7.7%), and 16 patients (4.7%), respectively. Most patients received antiviral therapy (276 [81.9%]) and antibacterial therapy (302 [89.6%]) during hospitalization. The proportion of treatment with the Chinese Medicine, glucocorticoid and immunoglobulin was 186 (55.2%), 150 (44.5%) and 56 (16.6%), respectively. Overall, the rates of these treatments had no significant differences in therapies between the group with and without hypertension. However, it is worth noting that hypertensive patients received treatment with glucocorticoid.

According to the diagnostic criteria, there were 73 (65.1%) patients with Grade I, 24 (21.4%) with Grade II and 15 (13.4%) with Grade III hypertension, respectively. All Grade III patients presented the severe or critical type of COVID-19. More than half of the patients with Grade III hypertension died ( Table 3 ).

**Table 3:** – The association between hypertension grade and disease severity in COVID-19 patients with hypertension

		Hypertension grade			

	Total (n=112)	I (n=73)	II (n=24)	III (n=15)	p-value
**Disease severity**					0.0003^a^
Moderate	20(17.8)	12(16.4)	8(33.3)	0(0)	
Severe	69(61.6)	50(68.5)	13((54.2)	6(40)	
Critical	23(19.3)	11(15.1)	3(12.5)	9(60)	
**Clinical outcomes**					0.0006^b^
Discharge	92(82.1)	64(87.7)	21(87.5)	7(46.7)	
Death	20(17.9)	9(12.3)	3(12.5)	8(53.3)	

*a: Kruskal-Wallis was used to analyze the relationship between disease severity and hypertension grades. b: R X C The Chi-square test was used to analyze the relationship between clinical outcomes and hypertension grades.*

Eighty-four (75%) hypertensive patients received antihypertensive treatment during hospitalization. Among them, 20 patients (17.8%) used angiotensin-converting enzyme inhibitors (ACEIs)/angiotensin receptor blockers (ARBs), and 64 (57.1%) received other antihypertensive drugs. The disease severity and clinical outcomes between the ACEI/ARB group and non-ACEI/ARB group did not present significant differences ( Table 4 ).

**Table 4:** – The association between ACEI/ARB use and disease severity in COVID-19 patients with hypertension

		Antihypertensive treatment			

	Total (n=112)	ACEI/ARB treatment (n=20)	Other hypotensive drug (n=64)	No hypotensive treatment (n=28)	p-value
**Disease severity**					0.3487^a^
Moderate	20(17.8)	3(15)	11(17.2)	6(21.4)	
Severe	69(61.6)	13(65)	36(56.3)	20(71.4)	
Critical	23(19.3)	4(20)	17(26.6)	2(7.1)	
**Clinical outcome**					1.0000^a^
Discharge	92(82.1)	16(80)	49(76.6)	27(96.4)	
Death	20(17.9)	4(20)	15(23.4)	1(3.6)	

*ACEIs: angiotensin-converting enzyme inhibitors; ARBs: angiotensin II receptor blockers. a: R X C Chi-square test was used to analyze the difference between groups.*

### Dynamic changes of levels during hospitalization

Since hypertensive patients presented higher levels of CRP, TnT, Creatinine, and ALT compared to normotensive patients, we further analyzed the dynamic change of these four laboratory markers during hospitalization among the surviving patients ( Figure 1 ). As shown in Figure 1A , the TnT level in hypertensive patients increased significantly during the course of hospitalization compared to non-hypertensive patients (median [IQR], 0.011 [0.008-0.021] vs. 0.008 [0.005-0.014], p=0.0027 at hospitalization and 0.012 [0.008-0.165] vs. 0.006 [0.005-0.012], p=0.0077 at approaching discharge). And no such dynamic escalation of TnT levels was observed in non-hypertensive patients. Likewise, the creatinine level in hypertensive patients increased significantly during the course of hospitalization compared to non-hypertensive patients (median [IQR], 69.0 [59.5-85.5] vs. 63.0 [51.3-75.8], p=0.0227 at hospitalization and 70.0 [59.0-84.0] vs. 64.0 [51.0-75], p=0.0220) at approaching discharge ( Figure 1B ).

**Figure 1:** – Dynamic change of TnT, Creatinine, CRP, and ALT during hospitalization. A.TnT; B. Creatinine; C.CRP; D.ALT. The data were expressed as the median and IQR. Mann-Whitney U test was used. (*p < 0.05, **p < 0.01, ***p < 0.001).

Both groups of patients exhibited high levels of CRP during the course of hospitalization. The CRP of non-hypertensive patients were controlled to normal range (median [IQR], 2.75[1.0-8.075]) with no significant differences comparing to the hypertensive group (median [IQR], 3.8[2.2-10.00]) at the time of approaching discharge ( Figure 1C ). Similarly, there were no significant differences in the ALT level between these two groups when approaching discharge ( Figure 1D ).

### Hypertension increases the death rate of patients with COVID-19

The relationship between hypertension and death was one of the focuses in our study. We found that mortality rates in hypertensive groups were higher than in normotensive groups. Meanwhile, hypertension was associated with nearly 2.2 more chances of dying due to COVID-19 (OR: 2.093 [95%CI: 1.094-4.006], p=0.024) according to the Chi-square test.

We further conducted a survival curve analysis using the Kaplan-Meier method. Patients with hypertension and those without hypertension had different survival curves during the time from admission to follow-up (mean=31,664, SED=1,424; mean=34.79, SED=0.882; p=0.0155) as shown in Figure 2A . Considering the duration of the illness at the time of admission, we also found that the survival curve of patients with hypertension and those without hypertension had no significant differences during the time from symptom onset to follow-up (mean=51,984, SE=2,703; mean=55,625, SE=2,139; p>0.05, Figure 2B ).

**Figure 2:** – *Kaplan–Meier plots of survival probability in hospitalized patients with COVID-19. A. Kaplan-Meier survival curves for mortality during the time from admission. B. Kaplan-Meier survival curves for mortality during the time from symptom onset.*

## Discussion

The world is currently suffering from an emerging infectious disease – COVID-19, which had 30,949,804 confirmed cases and 959,116 deaths until 21 September, 2020.^17^ Several studies demonstrated that hypertension has been the most common comorbidity in patients with COVID-19.^1 , 5^ In this cohort study, we provided detailed clinical characteristics and risk factors associated with clinical outcomes in COVID-19 hypertensive or normotensive patients. The overall case fatality rate in mainland China was 5.5% (4,642 deaths out of 84,393 confirmed cases on May 3, 2020).^17^ In our study, the prevalence of hypertension in COVID-19 patients was 33.2%, which is consistent with previous studies that reported the proportion of COVID-19 patients with hypertension ranging from 19.4 to 32.6%.^5 , 13 , 18^ The in-hospital mortality in patients with hypertension is markedly higher than in normotensive patients (17.9% vs. 8.9%, p=0.0202), in line with previous findings.

As we know, the angiotensin converting enzyme-2 (ACE-2), as an enzyme of the renin-angiotensin system (RAS), is the receptor for SARS-CoV-2 and essential for viral entry.^19^ ACE2 is not only expressed in type 2 alveolar epithelial cell in the lungs, but also in the renal tubules of the kidneys, cardiomyocytes in the heart, small intestinal epithelium in the gastrointestinal tract, bile duct epithelial cells and Leydig cells in the testis.^20^ Therefore, COVID-19 patients presented multiple extrapulmonary manifestations and possible complications. In our patient cohort, COVID-19 patients with hypertension had more comorbidities, such as diabetes, cardiovascular disease, liver disease, kidney disease and cerebrovascular disease. In this sense, hypertensive patients with COVID-19 presented abnormal levels of multiple indicators concerning the heart, liver, kidney, and lung function at hospital admission. Besides, COVID-19 patients with hypertension displayed higher levels of triglyceride and direct bilirubin. We also summarized other laboratory parameters that may be associated with worse progression of COVID-19 patients with hypertension. It is worth noting that hypertensive patients presented with significantly higher white blood cell count, neutrophil count, and lower lymphocyte count, which indicates that the level of lymphopenia is higher in COVID-19 patients with hypertension. It has been reported that lymphopenia is a common feature in patients with severe COVID-19, who display lower lymphocyte count, higher leukocyte count and neutrophil-lymphocyte ratio (NLR),^21^ with a dramatically reduced number of lymphocyte subsets and higher proinflammatory cytokine levels, including IL-2, IL-6, and IL-10.^22^ It is curious to determine if COVID-19 patients with hypertension also presented with severe dysregulation of the immune response compared to normotensive patients, but the surveillance of lymphopenia may be helpful in the treatment of COVID-19 patients with hypertension.

We further analyzed the dynamic changes of four biomarker levels during hospitalization, and found that ALT (liver injury) and CRP (inflammatory biomarkers) did not significantly differ between the hypertensive and the normotensive groups. Although the hypertension group displayed a slightly higher ratio of bacterial infection without statistical significance, we found that bacterial infection caused higher chances of death (OR: 5,867, 95%CI: 2,537-13,568, p<0.001). Nevertheless, hypertension was still a risk factor independently related to mortality after adjusting the effect of bacterial infection (OR: 2,029, 95%CI: 1,035-3,976, p<0.05), and the clinician should pay more attention to the secondary bacterial infection in the group with arterial hypertension regarding the higher levels of CRP. However, the TnT and creatinine levels of the hypertensive group were remarkable higher than those in the normotensive group during hospitalization and at the time of approaching discharge, which implies that more aggressive clinical management regarding cardiac and renal injury may be required for COVID-19 patients with hypertension. It was observed that the components of renin-angiotensin systems may play a pathogenic role for COVID-19 since ACE2 acts directly in hypertension and in SARS-CoV-2 transmission.^4^ The balance of the RAS pathway may determine the occurrence of tissue injury, especially in the heart and kidneys.^20^ Our data underlined the influence of hypertension on the severity of COVID-19, especially cardiac and kidney injury.

It is not surprising that COVID-19 patients with hypertension are experiencing higher frequency, severe forms, and more complications of COVID-19. Our further analyses found that the hypertension grade was associated with disease severity and clinical outcome in COVID-19 patients with hypertension. However, the mechanisms underlying the relationship between hypertension and COVID-19 are not well understood. As ACE2 acts as the receptor for SARS-CoV-2 to enter host cells, there are emerging concerns regarding the clinical use of ACEIs/ARBs, about whether or not these drugs could increase the susceptibility of a SARS-CoV-2 infection.^23^ Our data demonstrated that ACEIs/ARBs would not increase disease severity or the risk of death in COVID-19 patients with hypertension. Recently, a multi-center study including 1,128 COVID-19 patients with hypertension pointed out that the in-patient use of ACEI/ARB was associated with lower mortality in comparison with ACEI/ARB non-users.^14^ Combined with our results, these findings suggested that the continuous use of ACEI/ARB during hospitalization should be maintained to control the blood pressure for the patients’ benefit, since COVID-19 patients on ACEI/ARB were not at increased risk for adverse outcomes.

However, the present study has several limitations. Firstly, patients without major complications were assigned to the temporary treatment centers (mobile cabin hospitals) due to the limited medical resources, and all of the patients involved in this study had relatively severe cases of COVID-19. Secondly, the follow-up medical data were incomplete, as some critical cases were transferred to the ICU or the a superior hospital. These measures were conducted in accordance with national strategies for major epidemic prevention and control considering the emergency of the COVID-19 outbreak, which have great importance to mitigate the spread of the virus. Thirdly, only 20 patients received the ACEI/ARBs treatment, which may limit the determination of the potential use of ACEI/ARBs in COVID-19 treatment. Further clinical investigations are required.

## Conclusion

The present study suggested that hypertension has a significant association with disease severity and fatal outcomes of COVID-19. COVID-19 patients with hypertension presented with severe multiple organ manifestations and complications, especially myocardial and kidney injury, which implies that aggressive treatments may be considered for hypertensive patients diagnosed with COVID-19. Long-term observation and prospective study design on the effectiveness of treatments specific for COVID-19 patients with hypertension are required.
